# Increased multiple sclerosis disease activity in patients transitioned from fingolimod to dimethyl fumarate: a case series

**DOI:** 10.1186/s12883-021-02058-2

**Published:** 2021-02-02

**Authors:** Silvia Delgado, Jeffrey Hernandez, Leticia Tornes, Kottil Rammohan

**Affiliations:** Department of Neurology, MS Division, University of Miami Miller School of Medicine, 1120 NW 14 Street, Suite 1323, Miami, FL 33136 USA

**Keywords:** Multiple sclerosis, Fingolimod, Rebound, Dimethyl fumarate, Lymphopenia, Relapse, Case series, S1P receptor

## Abstract

**Background:**

Fingolimod is a S1P_1_ receptor modulator that prevents activated lymphocyte egress from lymphoid tissues causing lymphopenia, mainly affecting CD4+ T lymphocytes. Withdrawal from fingolimod can be followed by severe disease reactivation, and this coincides with return of autoreactive lymphocytes into circulation. The CD8+ T cytotoxic population returns prior to the regulatory CD4+ T lymphocytes leading to a state of dysregulation, which may contribute to the rebound and severity of clinical relapses. On the other hand, dimethyl fumarate (DMF) preferentially reduces CD8+ T lymphocytes, has the same efficacy as fingolimod, and therefore, was expected to be a suitable oral alternative to reduce the rebound associated with fingolimod withdrawal.

**Case presentation:**

We present six patients with relapsing-remitting MS who developed an unexpected increase in disease activity after transitioning from fingolimod to DMF. All patients were clinically and radiologically stable on fingolimod for at least 1 year. The switch in therapy was due to significantly low CD4+ T lymphocyte count ≤65 cells/ul (normal range 490–1740 cells/ul), after discussing the results with the patients and the potential risk for opportunistic infections including cryptococcal infections. DMF was introduced following a washout period of 5 to 11 weeks to allow reconstitution of the immune system and for the absolute lymphocyte count to reach ≥500 cells/ul. Every patient who experienced a relapse had several enhancing lesions in the brain and/or spinal cord between 12 to 23 weeks after cessation of fingolimod and 1 to 18 weeks after starting DMF. All relapses were treated with intravenous methylprednisolone with good clinical responses.

**Conclusion:**

The anticipated beneficial response of DMF treatment to mitigate rebound after fingolimod therapy cessation was not observed. Our patients experienced rebound disease despite being on treatment with DMF. Additional studies are necessary to understand which treatments are most effective to transition to after discontinuing fingolimod.

## Background

Fingolimod is an oral immunomodulatory agent approved to treat relapsing MS. It modulates S1P_1_ receptors blocking the egress of activated lymphocytes from lymph nodes with resultant lymphopenia, predominantly affecting CD4+ T cells [1, 2]. There are no specific guidelines for the monitoring of lymphocyte subsets of MS patients on fingolimod. However, several cases of cryptococcal infections have been reported in MS patients treated with fingolimod and low CD4+ T cell count [3–5]. Disease rebound within 6 months after fingolimod withdrawal has been documented [6–8], including several MS patients who were switched from fingolimod to dimethyl fumarate (DMF) [9]. The mechanism of rebound after fingolimod cessation is not well understood. Therefore, selection of a disease modifying therapy to prevent disease reactivation after fingolimod discontinuation remains a challenge in clinical practice.

## Case presentation

### Case 1

A 37-year-old woman diagnosed with MS in 2008 and treated with interferon-beta was transitioned to fingolimod in 2012 due to sub-optimal response. She remained clinically stable. In November 2017, she had sustained lymphopenia for 1 year with an absolute CD4+ T cell count of 33 cell/ul (normal range 490–1740 cells/ul). Fingolimod was discontinued in December 2017, and she started DMF in February 2018. Five weeks later, she developed weakness of both legs. MRIs brain and cervical spine showed several new enhancing lesions in the brain and one cervical at C2 level. She experienced another episode of walking difficulties and paresthesia in her legs in May 2018. MRI of brain showed 6 new enhancing lesions. Both episodes were treated with intravenous methylprednisolone with complete recovery.

### Case 2

**Fig. 1:**
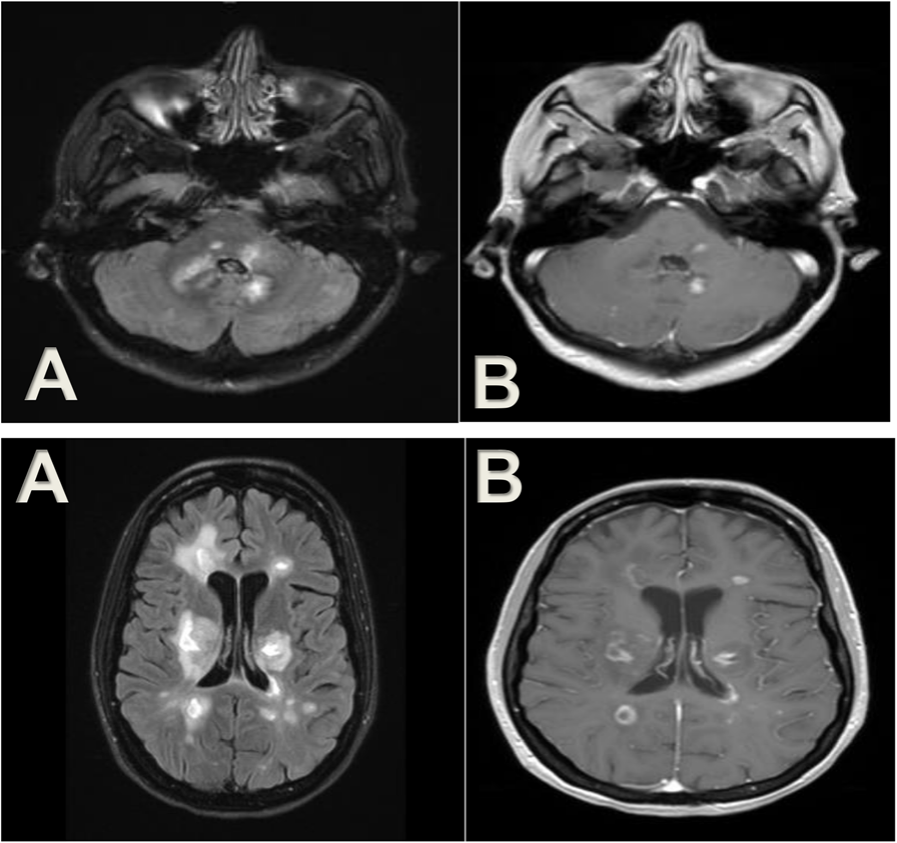
MRI of brain: **a**. Axial FLAIR **b**. Axial Post-Gadolinium. Patient had a severe MS relapse after fingolimod discontinuation for 12 weeks and on treatment with DMF for 4 days. MRI of brain showed multiple enhancing lesions with a nodular and ring enhancement pattern in the brainstem, posterior fossa, and cerebral hemispheres bilaterally (Case 2)


A 34-year-old woman diagnosed with MS in 2005 and treated with glatiramer acetate (GA) since 2010 was transitioned to fingolimod in 2013 due to sub-optimal response. Fingolimod was discontinued in December 2017 due to low CD4+ T cell count (< 20 cells/ul). Four days after starting DMF in mid-February 2018, she developed slurred speech, dizziness, paraparesis and ataxia. MRI studies showed several brain and cervical enhancing lesions at C2 and C4–5 levels. (Fig. 1) She was treated with intravenous methylprednisolone with good clinical response.

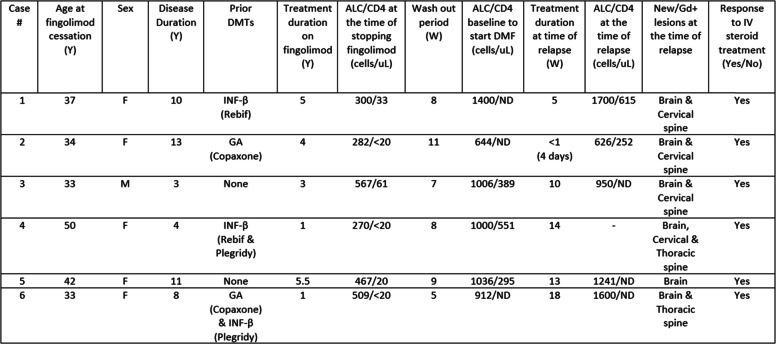


### Case 3

A 33-year-old man diagnosed with MS was started on fingolimod in 2014. He remained clinically stable. Fingolimod was stopped in December 2017 due to sustained low CD4+ T cell count (≤61 cells/ul), and DMF was started in February 2018. In mid-April 2018, he experienced tingling, first in the left hand followed by bilateral hands and feet, tightness in the abdomen above the waist bilaterally and perianal numbness. MRI of brain and cervical spine showed new enhancing lesions in the brain and within the cord at C4–5 level. Recovery was complete after treatment with intravenous methylprednisolone.

### Case 4

A 50-year-old woman diagnosed with MS in 2014 and treated with interferon-beta was switched to fingolimod in 2016 due to poor tolerability. Fingolimod was discontinued in November 2017 due to low CD4+ T cell count (< 20 cells/ul), and she was transitioned to DMF in January 2018. In mid-April 2018, she experienced right foot cramping, numbness/pain from her right breast down to her right foot for 2–3 weeks, with weakness of right leg, and walking difficulties. MRI of cervical and thoracic spine showed enhancing cord lesions at C4 and C7-T1 levels, within the right cerebellum, and at T9, T10, and T11 levels. She recovered well after intravenous methylprednisolone treatment. MRI of brain done after IV steroids showed three new non-enhancing lesions.

### Case 5

A 42-year-old woman diagnosed with MS in 2007 started treatment with fingolimod in 2012. In November 2017, fingolimod was discontinued due to sustained lymphopenia and low CD4+ T cell count (≤20 cells/ul). She was transitioned to DMF in January 2018. She complained of headache for 2 weeks and weakness of her legs in May 2018. Brain MRI showed four new lesions, three were enhancing. Treatment with intravenous methylprednisolone resulted in complete recovery.

### Case 6

A 33-year-old woman diagnosed with MS in 2010 treated with GA and then peg-interferon beta-1a, was switched to fingolimod in 2017 due to sub-optimal response. Fingolimod was discontinued in February 2018 due to lymphopenia and low CD4+ T cell count (< 20 cells/uL). She was transitioned to DMF in March 2018. In mid-July 2018, she experienced bilateral ascending numbness of the legs up to her hips over 2 days. MRIs demonstrated a new brain enhancing lesion and two new lesions in the thoracic cord, one of which was enhancing. She had a complete recovery after treatment with intravenous methylprednisolone.

## Results

Six relapsing-remitting MS patients (five women and one man), with a mean age of 38 years (range 33–50 years) at the time of fingolimod discontinuation, experienced increased disease activity with several enhancing lesions in the brain and/or spinal cord after being transitioned to DMF.

All the patients were treated with fingolimod for at least 1 year (range 1 to 5.5 years), and were clinically and radiologically stable prior to the transition in therapy. After a discussion with patients, a decision was made to switch treatments due to concerns for the risk of opportunistic infections including cryptococcal infections given the significantly low CD4+ T lymphocyte count [7, 8]. A wash out period (5 to 11 weeks) was done to allow the reconstitution of the immune system and for the absolute lymphocyte count (ALC) to return to ≥500 cells/ul prior to starting DMF. MS relapses were observed 12 to 23 weeks after fingolimod cessation and between 1 to 18 weeks after starting DMF. All relapses were successfully treated with intravenous methylprednisolone (Table 1).

**Table 1:** Case Summary

Case #	Age at fingolimod cessation (Y)	Sex	Disease Duration (Y)	Prior DMTs	Treatment duration on fingolimod (Y)	ALC/CD4 at the time of stopping fingolimod (cells/uL)	Wash out period (W)	ALC/CD4 baseline to start DMF (cells/uL)	Treatment duration at time of relapse (W)	ALC/CD4 at the time of relapse (cells/uL)	New/Gd+ lesions at the time of relapse	Response to IV steroid treatment (Yes/No)
1	37	F	10	INF-β (Rebif)	5	300/33	8	1400/ND	5	1700/615	Brain & Cervical spine	Yes
2	34	F	13	GA (Copaxone)	4	282/<20	11	644/ND	<1 (4 days)	626/252	Brain & Cervical spine	Yes
3	33	M	3	None	3	567/61	7	1006/389	10	950/ND	Brain & Cervical spine	Yes
4	50	F	4	INF-β (Rebif & Plegridy)	1	270/<20	8	1000/551	14	-	Brain, Cervical & Thoracic spine	Yes
5	42	F	11	None	5.5	467/20	9	1036/295	13	1241/ND	Brain	Yes
6	33	F	8	GA (Copaxone) & INF-β (Plegridy)	1	509/<20	5	912/ND	18	1600/ND	Brain & Thoracic spine	Yes

Absolute lymphocyte count (ALC): Normal reference range 850-3900 cells/ul (Quest Diagnostics)

Absolute CD4+ lymphocyte count: Normal reference range 490-1740 cells/ul (Quest Diagnostics)

*ND* Not done, *Y* Year, *W* Week, *Gd + Lesions* Gadolinium enhancing lesions

## Discussion and conclusions

CD4+ and CD8+ T lymphocytes play important roles in MS immunopathogenesis [10–13]. Expansion of T cell clones in active demyelinating MS brain lesions, showed a predominance of CD8+ T cells in all studied lesions, suggesting that these lymphocytes may be involved in auto-immune responses and cause tissue damage by cytotoxicity or cytokine release [11]. MS lesions may also contain regulatory CD4+ and CD8+ T cells that could halt the pathogenic processes, suggesting a dual and protective role of these cells [11, 13]. The balance between the immunomodulatory effects of these T cell subpopulations may be essential for disease remission.

The mechanism of rebound following discontinuation of fingolimod is unclear. It is postulated that the rebound may be mediated by the fast reappearance of previously entrapped autoreactive lymphocytes into the CNS [14]. Severe rebound after fingolimod discontinuation in mice with relapsing-remitting EAE was preceded by upregulation of S1P_1_ receptors in entrapped lymphocytes in lymph nodes followed by their egress into the circulation and subsequent CNS infiltration [15]. Mice that have a selective knockout of S1P_1_ receptors in their astrocytes developed attenuated EAE [16]. Astrocytic S1P_1_ overexpression after fingolimod cessation resulting in the release of inflammatory cytokines and nitric oxide may also contribute to MS rebound [17].

Another plausible hypothesis is that there is differential susceptibility of lymphocyte subsets to entrapment into secondary lymphoid organs, and an equally differential susceptibility for their return to circulation after withdrawal from fingolimod therapy. It is known that CD4+ T cells are most susceptible to entrapment, followed by CD8+ T cells [1, 2]. CD4+ T lymphocytes are the last to recover after withdrawal from fingolimod [18]. During this period of sequential return of lymphocytes, a dysregulated state may occur when autoreactive cytotoxic CD8+ T cells return earlier than the regulatory CD4+ T cells. The injury to the CNS may be mediated by the cytotoxic CD8+ T cells that remain unregulated. The injury may result in large tumefactive lesions in the brain, quite similar to the large lesions of acute disseminated encephalomyelitis (ADEM)-like event described in pediatric MS reported to be mediated by cytotoxic CD8+ T cells [19]. If the mechanism of CNS injury with the characteristic tumefactive lesions is primarily mediated by CD8+ T cells, DMF which is known to preferentially deplete the CD8+ T cell population [20] could be helpful in mitigating the fingolimod rebound phenomenon.

In our case series, the six patients experienced increased MS disease activity after transitioning from fingolimod to DMF, despite being on treatment, and one patient (Case 1) had a second relapse 3 months after starting DMF. This was an unexpected experience as DMF is an oral drug with an equivalent efficacy to fingolimod [21, 22]. Other authors have reported a similar experience with this transition strategy [9, 23]. It is likely that several immunopathogenic mechanisms are involved in MS disease reactivation after fingolimod cessation.

Limitations of this report include that it is a retrospective chart review, and the sample size is small and therefore, conclusions regarding treatment selection after fingolimod discontinuation cannot be drawn. This case series may help bring awareness to other providers who care for patients with MS of similar clinical scenarios. Further research is needed to determine the most effective treatment options after discontinuing fingolimod.

## Data Availability

All data related to this case report are contained within the manuscript. The first author can provide the original data if needed.

## Abbreviations

MS: Multiple sclerosis
DMF: Dimethyl fumarate
PML: Progressive multifocal leukoencephalopathy
S1P1: Sphingosine-1-phosphate receptor 1
MRI: Magnetic resonance imaging
GA: Glatiramer acetate
ALC: Absolute lymphocyte count
CNS: Central nervous system
EAE: Experimental autoimmune encephalomyelitis
ADEM: Acute disseminated encephalomyelitis
